# Supplementary material for: Attitudes and perceptions of undergraduate nursing students towards the nursing profession
Source: PLoS One. 2023 Jul 25;18(7):e0280700. doi: 10.1371/journal.pone.0280700 (PMC10368257; doi:10.1371/journal.pone.0280700)
Supplement: Attachment — Submitted filename: Response to Reviewers29042023.docx [file pone.0280700.s001.docx]

**PONE-D-23-00352 Choice, Attitudes, and Perceptions of Undergraduate Nursing Students towards the Nursing Profession**

**Response to Reviewers**

1. When submitting your revision, we need you to address these additional requirements. Please ensure that your manuscript meets PLOS ONE's style requirements, including file naming.

Response: These additional requirements have been addressed

2. Please provide additional details regarding participant consent. In the ethics statement in the Methods and online submission information, please ensure that you have specified what type you obtained (for instance, written or verbal, and if verbal, how it was documented and witnessed).

Response: Written consent was obtained. The Informed consent form had provision for the participants to acknowledge consent by appending a signature and the date.

3. Your ethics statement should only appear in the Methods section of your manuscript

Response: The ethics statement has been moved to the Methods selection on page 6.

4. Please review your reference list to ensure that it is complete and correct. If you have cited papers that have been retracted, please include the rationale for doing so in the manuscript text, or remove these references and replace them with relevant current references. Any changes to the reference list should be mentioned in the rebuttal letter that accompanies your revised manuscript. If you need to cite a retracted article, indicate the article’s retracted status in the References list and also include a citation and full reference for the retraction notice.

Response: The reference list has been reviewed

**Additional Editor Comments**:

This is well written paper that highlights choices, attitudes and perceptions of nursing students towards nursing profession. The methodology, especially for the qualitative section needs to be improved to include reliability, validity and trustworthiness.

Response: Reliability, validity and worthiness have been included in the qualitative methodology on page 6.

Are you sure a pilot study was done? If so, this is a mini study and their findings should also be published. What was the response rate? How many students were enrolled per class? In the methodology, preferably mention proportionate sampling , if it was done

Response: A pilot study was done. 12 students were enrolled, six per class however only four responded giving a response rate of 33.3%. I do not think their findings should be published.

Reviewer's Responses to Questions

Comments to the Author

Reviewer #1: The manuscript describes the context and approach of the study and is conducted in a rigorous manner. The data provided supports the conclusions. The statistical analysis if rigorously conducted and presentation is appropriate for the type of data being presented. The authors have provided a link for the data access for scrutiny. It is written in standard English with very few grammatical errors. The language is clear. The manuscript will contribute to this area of scientific research if published in the website. The authors have consulted a wide range of current works that has been publish to support the study and findings.

Reviewer #2: Dear authors, Thank you for giving me such great opportunity to review this manuscript. In general, the topic is interesting one and there is a much effort undertaken. I have some inquires and some comments that will strengthen your manuscript. I need to know why researchers conduct this study in Uganda. Numerous studies conducted and published concerning attitudes and perceptions of nursing students toward nursing profession. Also, factors shaping these attitudes and perceptions were also investigated too much. Results of the current study are the case of the vast majority of studies concerning attitudes and perceptions of students toward nursing profession. Is the context of nursing profession in Uganda is different?- what are the factors that make researchers to address this issue now in Uganda despite availability of solid background regarding attitudes and perceptions of different stakeholders of healthcare toward nursing profession.

Response: This study was conducted because of the low average enrolment of nursing students in Ugandan universities compared to other medical courses and incidences of nursing students changing study programs even after reaching third year of study.

The context of the nursing profession in Uganda is different, nursing students are equally important stakeholders and their views matter because they are directly associated with the profession compared to different stakeholders.

This manuscript is too long and need major revision. I know the effect of mixed research design in writing manuscript but this manuscript could be focused and summarized.

Response: The manuscript has been revised and shortened

Please follow the following comments;

**Abstract:**

- Sampling technique and timing of data collection need to be given.

Response: The sampling technique and timing have been included

**Introduction:**

- Context of nursing profession in Uganda should be reflected enough ( regulation of profession,

admission criteria, vital statistics, role of nursing in healthcare delivery system in Uganda)

Response: The context of the nursing profession in Uganda has been incorporated in the background

- What about the attitudes and perceptions of students toward nursing profession from other regions?

Response: The attitudes and perceptions of students from other regions has also been included

- Conceptual framework not clear( on which base researchers develop this framework)

Response: The conceptual framework has been omitted to shorten the manuscript. However it consisted of independent variables; age, gender, marital status, home area, year of study, institution of study, entry scheme, father’s education level and source of funding. The dependent variables were choice, attitudes and perceptions of undergraduate nursing students towards the nursing profession.

- Dependent and independent variables need to be addressed in introduction

Response: The independent variables have been addressed in the methods section, quantitative data collection methods and tools.

- I find results about determinants of attitudes of students toward nursing which is not reflected clearly in the aim of study

Response: The results have been omitted

- Why researchers ignore to add research question in this study?

Response: Research questions have been added

**Method:**

- Why researchers choose the purposive sampling to select participants of focus group?

Response: The participants chosen purposively were willing and able to provide relevant data to the study since they were knowledgeable about the topic

- Recruitment measures for participants should be mentioned - Sample representativeness, response rate is needed.

Response: Recruitment measures have been mentioned

- Timing of conducting the study should be stated

Response: Timing of conducting the study has been stated

- What are the measures used to ensure validity and trustworthy of data collected from subjects?

Response: To ensure validity, reliability and worthiness, the researcher provided clear information to the participants, established a relationship based on trust, accurately recorded and compared findings between the two groups

- What is the standard of focus group discussions; is it just point of view telling or data saturation?

Response: The standard was data saturation

- What about the structure of interview questions used in focus group discussion?

Response: A structured interview guide was followed

**Results:**

- Qualitative results need to be summarized ( many verbatim quotes not needed )

Response: Qualitative results have been summarized

- I feel if quantitative analysis used to reflect percent of students in each theme of attitudes yielded from focus group, it will give powerful impression since researchers depend only on 16 nurses to give us results about perceptions and attitudes which is questionable

Response: Quantitative analysis has been used to reflect percent of students in each theme

- Comparison of attitudes obtained from ASNP using mean scores did not give value to the readers. It is better to use mean score percent.

Response: This comment is not well understood

- What is the meaning of "relationship between students gender, marital status, religion, entry scheme and their attitudes was negligible but significant" ?

Response: This statement was irrelevant and has been omitted.

**Discussion:**

- It cannot find an interesting effective debate in discussion (researchers must keep their own voice too much using effective debate guidelines).

Response: Noted.

- Some results need to be discussed as why 6.1% only of students plan to join bedside nursing after graduation

Response: These results have been discussed

**Implications:**

- What are the implications from this study for both clinicians and healthcare leaders?

Response: Clinical instructors and lecturers should create awareness programs to equip students with knowledge about the nursing and its importance, focusing on improving the image of the profession.

Health care leaders should advocate for better remuneration and employment of nurses. They should also emphasize and embrace specialty in nursing to increase efficiency and improve quality of service delivery.
