# Supplementary material for: Attitudes and perceptions of undergraduate nursing students towards the nursing profession
Source: PLoS One. 2023 Jul 25;18(7):e0280700. doi: 10.1371/journal.pone.0280700 (PMC10368257; doi:10.1371/journal.pone.0280700)
Supplement: Attachment — Submitted filename: Comments plose.docx [file pone.0280700.s002.docx]

**Choice, Attitudes, and Perceptions of Undergraduate Nursing Students towards the Nursing** Profession

**Review**

**Comments to the Author**

**I noticed major issues that needs to be concerned**

**Title**

The Title should be modified as remove as choice, “Attitudes and Perceptions of Undergraduate Nursing Students towards the Nursing Profession “ because they are already engagement of the profession.

**Abstract**

**Background**: please try to describe precise information about perception and attitude and finaly show the gap here

**Methods**: you describe only Attitude but what about perception related questionnaires, and please incorporate data analysis information in methodology part

**Result**: first write the prevalence then significant variable. what is your justification:- poor academic performance in high school, desire to pursue a medical course, failure to get admitted for Medicine and Surgery, low cut-off points for the course and interest in nursing.

Attitude result shows 81.8% what about perception and choice?

**Conclusion**: totally your conclusion wrong <pre-nurse counseling and introductory courses about the profession to enhance the acquisition and maintenance of an accurate perception of the nursing profession> they discuss not being included in your finding. Add please concise and short recommendation in the conclusion.

**Background**

Please describe in detail factors magnitude of attitude, perception in background.

**Methods**

Please replace study setting with study setting and **period**

Despite you used a self-administered survey that took two months to complete, your sample of 165 people is genuine

**Study participants**

Please write in the form of source population and study population than study participants.

**Sampling strategy**

- Please write sampling strategy in clear and concise way. In general it is not clear the strategy
- I'm not understanding how to take 160 samples please write with evidence based .
- Your sample sizes are not adequate and dose not representative this small size sample. So it is difficult to accept your finding for scientific evidence purpose.
- It is even possible to include other national-level universities.

**Sampling procedure**

Please write clearly show your sampling procedure in clear and specific schematic way

**Quantitative data collection**

For attitude type use a five-point Likert-type but what about perception.

Please write attitude and perception tools in a separated and clearly way

The response rate was 91.6%; what had happened prior to these results?

Your Pilot study samples size not be correct. Because 15% of your sample is12 replace by 24

**Quantitative data analysis**

Completely unclear data analysis procedure; please rewrite again; it would be wiser to start with a clean check and enter and so…..

**Result**

First the prevalence then the response rate in the result?

It is better to write majority 89.1% Christian and Busitema University students around half like (50%) and so….

Marital status and religion should have more than two add others (specify)

Father education level **tertiary** it need operationalized

Overall, the respondents perceived nursing as a good and enjoyable **course (nursing is profession or course) not clear to me**

**Discussion**

The first paragraph no need of write the objective here it is better to write general information about attitude and perception.

In the second paragraph these results are in agreement with a similar study in choice what is your justification in general your result look like possibility not real finding.

**Attitudes of nursing students towards the nursing profession**

The mean attitude score was 129.9±12.3 (minimum 87, maximum 153).but in data collection show that It is a five-point Likert-type scale with a minimum score of 40 and maximum of 200)

Before compare your result first writes the prevalence and range of attitude

Your study prevalence 81.8% had positive attitudes towards the nursing profession then how 62.07% similar **Hue University of Medicine and Pharmacy need clarification**

**In general the whole discussion need detail description compare and contract**

**Conclusion**

Your conclusion part not be focus your result. Please avoid terms like receive pre-nurse counseling and introductory courses about the profession, to enhance acquisition and maintenance of an accurate perception related variable, and conclude in line with your finding.

**Limitations to the study**

The findings cannot be generalized private but also the governmental nursing institutions in Uganda, because your sample size is very small.

**Implications of this study**

In general the implication of your study not based on your results

Reference

Most of the reference out dated eg Ref 14,15,23 ….

In general, I am interested in paper and recommend publication with major modifications.
