# Supplementary material for: Attitudes and perceptions of undergraduate nursing students towards the nursing profession
Source: PLoS One. 2023 Jul 25;18(7):e0280700. doi: 10.1371/journal.pone.0280700 (PMC10368257; doi:10.1371/journal.pone.0280700)
Supplement: Attachment — Submitted filename: Response to Reviewers05072023.docx [file pone.0280700.s003.docx]

Choice, Attitudes, and Perceptions of Undergraduate Nursing Students towards the Nursing Profession Review

| Comments | Response to comments | Reference page |
| --- | --- | --- |
| Title  The Title should be modified as remove as choice, “Attitudes and Perceptions of Undergraduate Nursing Students towards the Nursing Profession “ because they are already engagement of the profession. | The title has been modified to remove choice | Page 1 |
| Abstract  Background: please try to describe precise information about perception and attitude and finally show the gap here | The perception and attitudes have been described precisely | Page 1 |
| Methods: you describe only Attitude but what about perception related questionnaires, and please incorporate data analysis information in methodology part | The ASNP is used to measure attitudes towards the nursing profession, it does not measure perceptions and choice. |  |
| Result: first write the prevalence then significant variable. what is your justification:- poor academic performance in high school, desire to pursue a medical course, failure to get admitted for Medicine and Surgery, low cut-off points for the course and interest in nursing.  Attitude result shows 81.8% what about perception and choice? | The results for perceptions are qualitative | Page 1 |
| Conclusion: totally your conclusion wrong <pre-nurse counseling and introductory courses about the profession to enhance the acquisition and maintenance of an accurate perception of the nursing profession> they discuss not being included in your finding. Add please concise and short recommendation in the conclusion. | A concise recommendation has been added to the conclusion | Page 1 |
| Background  Please describe in detail factors magnitude of attitude, perception in background. | This has been considered | Page 2 |
| Methods  Please replace study setting with study setting and period  Despite you used a self-administered survey that took two months to complete, your sample of 165 people is genuine | Study setting has been replaced with study setting and period  The sample size was the total number of nursing students in the two universities. | Page 4 |
| Study participants  Please write in the form of source population and study population than study participants. | Study participants has been replaced with study population | Page 4 |
| Sampling strategy  Please write sampling strategy in clear and concise way. In general it is not clear the strategy  I'm not understanding how to take 160 samples please write with evidence based.  Your sample sizes are not adequate and dose not representative this small size sample. So it is difficult to accept your finding for scientific evidence purpose.  It is even possible to include other national-level universities | The sample size was the total number of nursing students in the two universities.  All the students were invited to respond to the questionnaire because of the small number. | Page 4 |
| Sampling procedure  Please write clearly show your sampling procedure in clear and specific schematic way | For the quantitative arm, all students were invited to fill the questionnaire, they were not sampled  Purposive sampling was used for the Focus Group Discussions only | Page 4 |
| Quantitative data collection  For attitude type use a five-point Likert-type but what about perception.  Please write attitude and perception tools in a separated and clearly way  The response rate was 91.6%; what had happened prior to these results?  Your Pilot study samples size not be correct. Because 15% of your sample is12 replace by 24 | Perceptions were collected through the Focus Group Discussions. They were qualitative findings of the study  Pilot study sample size has been corrected | Page 5 |
| Quantitative data analysis  Completely unclear data analysis procedure; please rewrite again; it would be wiser to start with a clean check and enter and so….. | The data analysis procedure has been clearly described | Page 6 |
| Result  First the prevalence then the response rate in the result?  It is better to write majority 89.1% Christian and Busitema University students around half like (50%) and so….  Marital status and religion should have more than two add others (specify)  Father education level tertiary it need operationalized  Overall, the respondents perceived nursing as a good and enjoyable course (nursing is profession or course) not clear to me | The first section is simply describing the socio-demographics of the participants  There was no other religion noted in the responses, except denominations of Christianity such as Anglicans and Catholics  Nursing is a profession not a course |  |
| Discussion  The first paragraph no need of write the objective here it is better to write general information about attitude and perception.  In the second paragraph these results are in agreement with a similar study in choice what is your justification in general your result look like possibility not real finding.  Attitudes of nursing students towards the nursing profession  The mean attitude score was 129.9±12.3 (minimum 87, maximum 153).but in data collection show that It is a five-point Likert-type scale with a minimum score of 40 and maximum of 200)  Before compare your result first writes the prevalence and range of attitude  Your study prevalence 81.8% had positive attitudes towards the nursing profession then how 62.07% similar Hue University of Medicine and Pharmacy need clarification  In general the whole discussion need detail description compare and contract | The minimum score one can obtain using the ASNP is 40 and the maximum is 200 but for this study, the minimum score obtained was 87 and the maximum was 153. |  |
| Conclusion  Your conclusion part not be focus your result. Please avoid terms like receive pre-nurse counseling and introductory courses about the profession, to enhance acquisition and maintenance of an accurate perception related variable, and conclude in line with your finding. | The conclusion has been improved | Page 15 |
| Limitations to the study  The findings cannot be generalized private but also the governmental nursing institutions in Uganda, because your sample size is very small. | This limitation has been included | Page 15 |
| Implications of this study  In general the implication of your study not based on your results  Reference  Most of the reference out dated eg Ref 14,15,23 ….  In general, I am interested in paper and recommend publication with major modifications. | Reference 14 is The Uganda Nurses and Midwives Act,1996 which provides for the training, registration, enrollment and discipline of nurses and midwives in Uganda. It was enacted in 1996 and has not been reviewed since then.  Reference 23 is the Attitude Scale for the Nursing Profession which was developed in 2011. |  |
